# Perifosine, a Bioavailable Alkylphospholipid Akt Inhibitor, Exhibits Antitumor Activity in Murine Models of Cancer Brain Metastasis Through Favorable Tumor Exposure

**DOI:** 10.3389/fonc.2021.754365

**Published:** 2021-11-04

**Authors:** Keisuke Taniguchi, Tomo Suzuki, Tomomi Okamura, Akinobu Kurita, Gou Nohara, Satoru Ishii, Shoichi Kado, Akimitsu Takagi, Momomi Tsugane, Yoshiyuki Shishido

**Affiliations:** ^1^ Yakult Central Institute, Yakult Honsha Co., Ltd., Tokyo, Japan; ^2^ Pharmaceutical Research & Development Department, Yakult Honsha Co., Ltd., Tokyo, Japan

**Keywords:** brain cancer, metastasis, signaling pathway, PI3K, AKT, apoptosis

## Abstract

Metastatic brain tumors are regarded as the most advanced stage of certain types of cancer; however, chemotherapy has played a limited role in the treatment of brain metastases. Here, we established murine models of brain metastasis using cell lines derived from human brain metastatic tumors, and aimed to explore the antitumor efficacy of perifosine, an orally active allosteric Akt inhibitor. We evaluated the effectiveness of perifosine by using it as a single agent in ectopic and orthotopic models created by injecting the DU 145 and NCI-H1915 cell lines into mice. Initially, the injected cells formed distant multifocal lesions in the brains of NCI-H1915 mice, making surgical resection impractical in clinical settings. We determined that perifosine could distribute into the brain and remain localized in that region for a long period. Perifosine significantly prolonged the survival of DU 145 and NCI-H1915 orthotopic brain tumor mice; additionally, complete tumor regression was observed in the NCI-H1915 model. Perifosine also elicited much stronger antitumor responses against subcutaneous NCI-H1915 growth; a similar trend of sensitivity to perifosine was also observed in the orthotopic models. Moreover, the degree of suppression of NCI-H1915 tumor growth was associated with long-term exposure to a high level of perifosine at the tumor site and the resultant blockage of the PI3K/Akt signaling pathway, a decrease in tumor cell proliferation, and increased apoptosis. The results presented here provide a promising approach for the future treatment of patients with metastatic brain cancers and emphasize the importance of enriching a patient population that has a higher probability of responding to perifosine.

## Introduction

The development of metastases adversely affects both quality of life and survival, and 20–40% of all cancer patients eventually experience brain metastasis (1). Any type of tumor can spread to the brain; however, the types of cancer that are most likely to metastasize to the brain are lung, breast, colorectal, renal, and melanoma, whereas gastrointestinal and prostate cancers are less frequent (2). Despite recent progress in the field of molecular targeted therapy, strategies to effectively treat metastatic brain tumors remain insufficient (3). Consequently, an urgent need remains for the development of effective therapies for these patients. Brain tumors, regardless of whether they are primary or metastatic, are difficult to control, and curative surgery frequently is not a feasible option. Most anticancer agents have limited access to the central nervous system (CNS) following systemic administration. One of the primary causes of this is the blood-brain barrier (BBB) that leads to relatively ineffective drug concentrations within the CNS tissue, a characteristic of a number of chemotherapeutic drugs. This, in turn, hinders the antitumor efficacy of these drugs in the brain following local or systemic administration (4). Based on this, antineoplastic agents that can penetrate the CNS and achieve long-term and high-level exposure are likely to be effective in the treatment of primary and metastatic brain tumors.

PI3K/Akt signaling has been implicated in various malignant cancers and is one of the key signal transduction cascades involved in the control of cellular proliferation, invasion, and survival (5–12). Akt is acknowledged as a major downstream effector of PI3K; Akt becomes fully activated through its phosphorylation at both T308 and S473 (6, 7). Constitutive Akt activation has been demonstrated to render tumor cells highly invasive (5). Additionally, Akt activation has been reported to induce epithelial-to-mesenchymal transition by regulating matrix metalloproteinases, ultimately resulting in increased invasiveness and metastasis (5, 13, 14). Thus, the PI3K/Akt pathway is considered as an attractive target for cancer therapy, and several inhibitors targeting this pathway are currently under evaluation in preclinical and clinical studies (5, 15, 16). Perifosine is the first orally bioactive alkylphospholipid that is currently being tested in clinical trials (16–19). Although the specific mechanisms underlying the anti-cancer activity of perifosine remain to be fully elucidated, perifosine is known to bind to the pleckstrin-homology domain that targets Akt activity by perturbing the membrane translocation of Akt (17–20).

To date, the potential role of perifosine in the context of metastatic brain cancers has not been thoroughly examined. Herein, we sought to evaluate the usefulness of human metastatic brain tumor models, using tumor cell lines originating from human brain metastatic sites, to assess the effectiveness of perifosine against these tumor models. Our results indicated that orally administered perifosine was efficiently delivered into tumor tissues and exhibited powerful antitumor efficacy. Our findings support the concept that prolonged exposure to a high level of perifosine at the tumor site and the suppression of Akt pathway activation are both vital events underlying *in vivo* antitumor activity. Given these encouraging findings, perifosine appears to be a promising candidate for the future treatment of patients with metastatic brain cancers.

## Materials and Methods

### Human Cancer Cell Lines

The primary glioblastoma cell line U-87 MG, the hormone-refractory prostate cancer cell line DU 145, and the large cell lung cancer cell line NCI-H1915 (hereinafter “H1915”) were purchased from the American Type Culture Collection (ATCC, Manassas, VA). DU 145 and H1915 cell lines were originally isolated from a brain metastasis. All cell lines were maintained according to the supplier’s instructions and routinely tested for mycoplasma contamination using MycoAlerTM (Lonza, Walkersville, MD, USA).

### Chemicals, Antibodies, and Reagents

Perifosine was obtained from Aeterna Zentaris GmbH (Frankfurt, Germany). For the *in vitro* assays, perifosine was dissolved in dimethyl sulfoxide (DMSO), and the final concentration of DMSO was adjusted to 0.1%. For the *in vivo* studies, perifosine was dissolved in physiological saline (FUSO Pharmaceutical Industries, Ltd., Osaka, Japan). The antibodies used in this study are listed in **Supplementary Table 1**. EnVision System HRP-Labelled Polymer Secondary antibody was purchased from Agilent Technologies (Santa Clara, CA, USA). All other reagents were obtained from Sigma-Aldrich (St. Louis, MO, USA) unless otherwise specified.

### Dose Response Curves for IC_50_ Determination

Dose response curves were generated for perifosine to determine the inhibitory concentration required to achieve 50% cell death (IC_50_ values). Briefly, cells were grown overnight in 96-well plates and then left untreated or treated with perifosine at different concentrations. After 48 h, the extent of cell viability was assessed according to a WST-8 dye-based assay (Kishida Chemical, Osaka, Japan) as described previously (21). For each treatment condition, the mean values from triplicate wells were calculated.

### Development of Tumor Xenograft Models

Five-week-old male BALB/c nude mice were purchased from Japan SLC, Inc. (Shizuoka, Japan).

#### Protocol Number 1 (Intracranial Tumor Transplantation)

Brain tumors were initiated using the cerebral injection procedure. Tumor cells can either be injected “freehand” or through the use of stereotactic instruments. The hole in the skull can be created either by simply pricking with a two-step injection needle (tip: 27G, 3 mm; Natsume Seisakusyo, Tokyo, Japan) or through the use of a small drill (1 mm anterior and 2 mm to the right of the bregma) (22–27). We preliminarily confirmed that all intracranial orthotopic brain metastasis mice created using U-87 MG, DU 145, and H1915 cells, but none of the sham-operated mice, exhibited abnormalities such as emaciation, falling, ataxic gait, and/or stupor over time, and all mice reached an endpoint when they were not treated, regardless of any differences between the two procedures (n = 3–15). Based on these findings, we selected the freehand method owing to our need to inoculate large numbers of mice, as this method increases throughput relative to the use of the stereotactic frame. Briefly, mice were anesthetized by inhalation of 1.5% isoflurane (Mylan Seiyaku Ltd., Tokyo, Japan). The dorsal head surface of mice was disinfected with 70% alcohol. A 3 mm two-step injection needle attached to a microsyringe (Hamilton Company, Reno, NV, USA) was inserted into a unilateral injection site to create a hole in the skull, and the needle was inserted intracranially at 3 mm below the skull surface. U-87 MG (1 × 10^6^), DU 145 (5 × 10^5^), or H1915 (7 × 10^5^) cells suspended in 5 μL PBS were then injected (day 0). The sham-operated mice underwent the same procedures but did not receive tumor cell injections.

#### Protocol Number 2 (Subcutaneous Tumor Transplantation)

DU 145 (7 × 10^6^ cells in 0.1 mL) or H1915 (3 × 10^6^ cells in 0.1 mL) cells were subcutaneously inoculated into the right flank of mice and allowed to form a palpable tumor.

### Evaluation of Antitumor Activity

We designed a loading (180 mg/kg) and maintenance (45 mg/kg) dosing regimen for a series of experiments, with the aim of efficiently delivering perifosine into tumor sites. The chemotherapy experiments were performed using two different protocols as described below.

#### Protocol Number 1 (Intracranial Orthotopic Brain Metastasis Models)

Prior to the initiation of efficacy studies, we preliminarily examined tumor growth at the injection site to provide a rationale for treatment schedules, histologically detected viable tumor cells at three days after tumor cell injection (day 3), and determined the characteristics of growth tendencies on day 7. Although therapy is often initiated as early as within two days of inoculation owing to rapidly progressive diseases in preclinical models (28–30), we selected “day 3” or “day 7” as the initial day of treatment based on this preliminary result and in anticipation of clinical use of perifosine.

The animals were assigned homogeneously to each test group based on the body weight on day 3. The animals were treated orally with either vehicle or perifosine with a 5-day-on/2-day-off schedule, as indicated in **Tables 1** and **2**. The survival times of the mice were then tested for 64 days. For humane reasons, the mice were euthanized when they appeared moribund, and this time point was defined as death in our survival analysis. Mortality was monitored by recording the percentage increase in life span (ILS) and median survival time (MST) according to the following formula: ILS (%) = (MST of the treated group/MST of the control group – 1) × 100. On day 64, blood was collected from the abdominal vena cava of survived mice under isoflurane anesthesia at 24 h after the final dose. Blood samples were treated with heparin to obtain plasma. Plasma levels of glucose, total cholesterol (T-CHO), total bilirubin (T-BIL), blood urea nitrogen (BUN), creatinine (CRE), alkaline phosphatase (ALP) were analyzed using an automatic chemistry analyzer (LABOSPECT003, Hitachi High-Tech Co., Tokyo, Japan).

**Table 1:** Group configuration in the DU 145 intracranial orthotopic brain metastasis model.

Group	Start of administration	Dosing article	Dose (mg/kg)	No. of animals
Control	day 3	Saline	0	5
Perifosine (D3)*	day 3	Perifosine	180/45	5

*180 mg/kg loading dose on day 3, followed by maintenance doses of 45 mg/kg.

**Table 2:** Group configuration in the H1915 intracranial orthotopic brain metastasis model.

Group	Start of administration	Dosing article	Dose (mg/kg)	No. of animals
Control	day 3	Saline	0	10
Perifosine (D3)*	day 3	Perifosine	180/45	10
Perifosine (D7)*	day 7	Perifosine	180/45	10

*180 mg/kg loading dose on day 3 or 7, followed by maintenance doses of 45 mg/kg.

#### Protocol Number 2 (Subcutaneous Ectopic Tumor Models)

Day “n” denotes the day on which the effects of drugs were estimated, and day “1” denotes the first day of treatment. Once the tumors reached an average volume of 100 mm^3^, the animals (n = 5) were randomly allocated into two groups based on tumor volume (TV) and then administered orally with either vehicle or a perifosine (180 mg/kg) *loading* dose on day 1 followed by *maintenance* doses of 45 mg/kg (180/45) with a 5-day-on/2-day-off × 3 cycle schedule. Tumor growth was monitored until day 22 by measuring two perpendicular diameters with a digital caliper (Mitutoyo, Kanagawa, Japan), and TV was calculated as shown previously (31, 32). The tumor growth inhibition rate (TGI, %) was calculated using the following formula:


$$\begin{array}{l} \text{TGI}\left(\%\right)=c(1\;–\;\text{mean}\;\text{TV}\;\text{of}\;\text{the}\;\text{perifosine}\\ \;–\text{treated}\;\text{group}/\text{mean}\;\text{TV}\;\text{in}\;\text{the}\;\text{control}\;\text{group})\;\times \;100 \end{array}$$

On day 22, xenograft tumors were excised, weighed, and snap-frozen at 4 h following the last dose. The body weight of each mouse was monitored twice each week to assess the systemic toxicity of this therapy. The relative body weight (RBW) at day n was calculated using the following formula:

RBW = body weight on day n/body weight on day 1

### Pathological Analysis

Brain or subcutaneous tumors were fixed in 10% formalin for approximately 72 h, embedded in paraffin, and sectioned at 4 µm thickness. Sections were stained with hematoxylin and eosin (H&E) for morphological observations, and with van Gieson for detection of fibrosis using a standard procedure. For immunostaining, the sections were preincubated with 3% H_2_O_2_ to block endogenous peroxidases. Sections were then incubated with antibodies against cytokeratin (clone AE1/AE3) and Ki-67 (clone SP6) overnight at 4°C and cleaved caspase-3 for 60 min at room temperature. After rinsing in Tris buffer, the secondary antibody was applied for 30 min at room temperature, and this was followed by the addition of 3,3’-diaminobenzidine (DAB) as a substrate. The sections were then counterstained with hematoxylin. Ki-67, cleaved caspase-3, and van Gieson-stained sections were graded by a pathologist (TS) as follows: -, absent; ±, minimal; 1+, mild; and 2+, moderate (**Supplementary Figure 1**).

### Mechanistic Analysis of Antitumor Effects

Tumor lysates were prepared from mice in each group on day 22 in *Protocol Number 2 (subcutaneous ectopic tumor models)*. Western blot analysis of the phosphorylation/activation patterns of the relevant molecules was performed as previously described (21). All the western blots were normalized to β-actin, and protein intensity was quantified using the Image J software (NIH, Bethesda, MD).

### Pharmacokinetics of Perifosine

Non-tumor-bearing mice were orally administered a single dose of perifosine at 45 or 180 mg/kg. At 4, 8, 24, 96, 168, and 360 h after the dose, blood was collected from the abdominal vena cava under isoflurane anesthesia, and the brain was resected after exsanguination. In a separate set, the DU 145 and H1915 subcutaneous ectopic tumor models were administered perifosine as either a single dose (45 or 180 mg/kg) or repeated doses (180 mg/kg *loading* dose followed by four consecutive daily doses of 45 mg/kg [180/45]). At 24, 48, 96, 144, and 168 h after the first dose, blood and tumor tissues were obtained in a manner similar to that described above.

The plasma was mixed with an equal volume of 1% formic acid and stored at -80°C. It (10 μL) was then mixed with an equal volume of acetonitrile containing 1% formic acid (FA/ACN) and 180 μL of internal standard (IS) solution (200 ng/mL of ethyl *p*-hydroxybenzoate [EHB] solution in FA/ACN). Next, the supernatant was collected after deproteinization (Sirocco, Waters Corp.) for LC-MS/MS assay analysis. The brain and tumor tissues were mixed with an equal volume (w/v) of 1% formic acid, homogenized, mixed with a 3-fold volume of FA/ACN, and centrifuged at 15,000 rpm (16,617 × g) for 3 min at 4°C to collect the supernatant. The supernatant (20 μL) was mixed with 80 μL of IS solution (200 ng/mL of EHB solution in “FA/ACN”/water [3:1] solution), and the new supernatant was obtained after deproteinization using the LC-MS/MS assay. The maximum plasma drug concentration (C_max_), the area under the concentration-time curve up to the last sampling time-point (AUC_0-last_), the mean residence time (MRT), and the terminal half-life (T_1/2_) were calculated using the mean values at each point. The AUC_0-last_ was calculated using the trapezoidal rule. The MRT was calculated as follows: $\text{MRT}\;=\;\text{AUMC}/\text{AUC}\;\left(\text{AUMC}:\;\text{area}\;\text{under}\;\text{moment}\;\text{curve}\;=\int_{\text{last}}^{\infty }\text{tC}dt\right)$ .

### Statistical Analysis

Data were analyzed using SAS System Release 8.2 (SAS Preclinical Package, Version. 5.0; SAS Institute Japan Ltd., Tokyo, Japan). The values are expressed as mean ± standard deviation (SD). Differences in survival among experimental groups were analyzed according to Kaplan-Meier survival curves using the log-rank test. The Student’s *t*-test or Welch’s *t*-test (F-test; *p* < 0.05) was used to detect the statistical differences in brain weight, TV, tumor weight, RBW, and protein levels in tumor tissues between two groups. P values < 0.05 were considered to be statistically significant. 

## Results

### Brain Tissue Distribution Profiles of Perifosine

**Figure 1:**
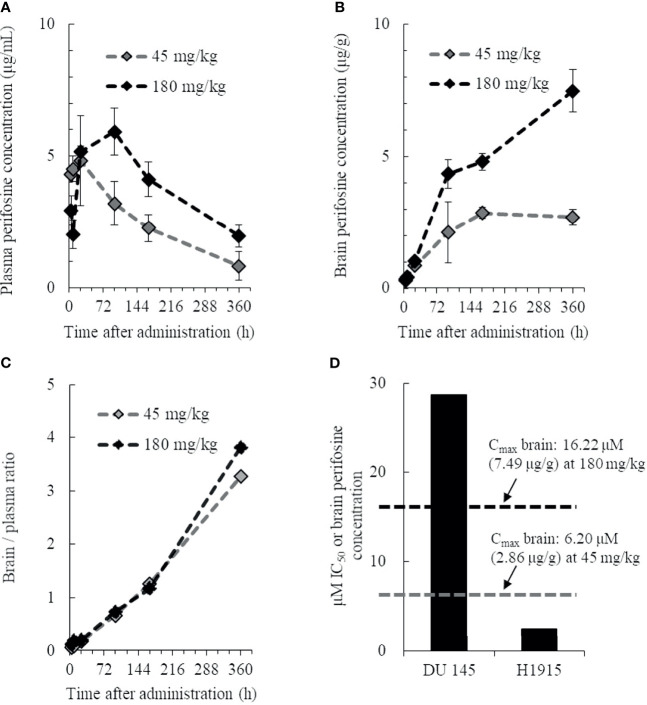
Pharmacokinetic profile of perifosine in plasma and brain tissue. Healthy animals were administered a single oral dose of perifosine, and at six intervals, mice from each group were then euthanized for plasma **(A)** and brain **(B)** extraction (n = 3). **(C)** The brain-to-plasma ratio of perifosine was calculated at each time point. **(D)** Relationship between the brain C_max_ and *in vitro* IC_50_ for DU 145 and H1915 cells.

To investigate whether perifosine is extensively distributed in brain tissues, we examined the plasma and brain tissue levels of perifosine in normal mice with intact BBBs. Perifosine was administered at doses of 45 or 180 mg/kg, as the maximum tolerated dose of perifosine was confirmed to be 45 mg/kg (once-daily regimen) and 250 mg/kg (once-weekly regimen). The plasma perifosine concentrations reached maximum levels at 24 or 96 h post-dosing and exhibited a gradual decline thereafter (**Figure 1A**). In contrast, brain concentrations increased gradually and remained at high levels for at least up to 360 h (Brain C_max_: 6.20 μM [2.86 μg/g] for 45 mg/kg and 16.22 μM [7.49 μg/g] for 180 mg/kg, respectively) (**Figure 1B**). High and rapid distribution in brain tissues was observed in the 180 mg/kg group compared to that observed in the 45 mg/kg group (**Figure 1B**). When calculating the brain-to-plasma ratios for perifosine, a trend toward a higher retention in brain tissues was observed at 360 h post-dosing (**Figure 1C**); however, this was not observed at the earlier time-points. The *in vitro* perifosine IC_50_ values for DU 145 and H1915 were 28.8 μM and 2.5 μM, respectively, at 48 h; these were approximately 1.8-fold higher and 6.5-fold lower, respectively, than the brain C_max_ values after a single oral dose of 180 mg/kg (**Figure 1D**).

### Growth Characteristics of Tumor Cell Lines in Intracranial Orthotopic Brain Metastasis Models

**Figure 2:**
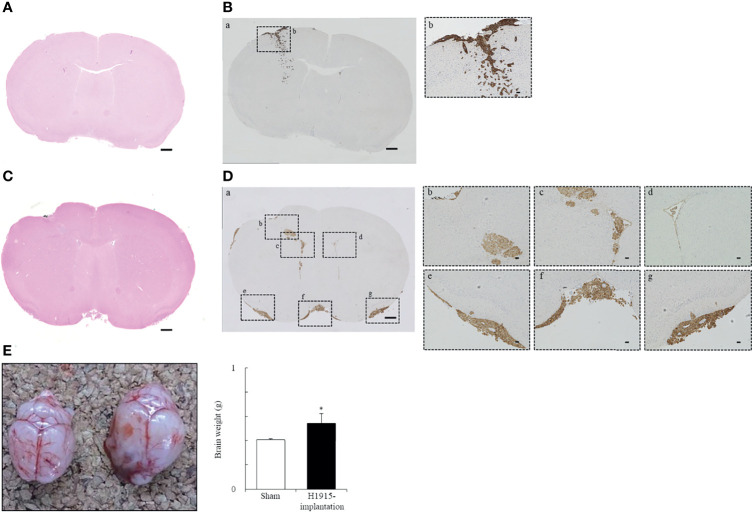
Representative morphological features of tumor cell lines following implantation. **(A–D)** Histological and **(E)** macroscopic images of the brains. Intracranial injection of DU 145 **(A, B)** and H1915 **(C, D)** cells into nude mice shows characteristic tumor growth patterns in brain on day 14. Representative H&E-stained **(A, C)** and cytokeratin (AE1/AE3)-stained **(Ba, Da)** sections are shown (scale bar = 500 μm). Boxed areas in panels **(Ba)** and **(Da)** show regions depicted in panels **(Bb)** and **(Db-g)**, respectively. Cytokeratin (AE1/AE3) were used to distinguish brain tissues from metastatic DU 145 **(Bb)** and H1915 **(Db-g)** tumors; scale bar = 50 μm. **(E)** Gross features of representative brains excised from sham-operated (left; n = 5) and H1915-implanted (right; n = 4) mice on day 21. *p < 0.05 versus the sham-operated group.

Intracranial inoculations were performed using human primary glioblastoma U-87 MG cells and two human metastatic brain tumor cell lines. Of these cell lines, U-87 MG cells formed a unifocal, unilateral tumor in the cerebral parenchyma, which was consistent with previous reports (23, 33) (**Supplementary Figure 2**). Injection with DU 145 cells caused scattered formation of small tumor nests in the cerebral parenchyma near the injection site. These multiple tumors were found separately (**Figures 2A, Ba, b**), unlike a solitary lesion induced by U-87 MG cells. Injection of H1915 cells resulted in multifocal tumors. Tumor cells were observed not only in the brain parenchyma but also in the subarachnoid spaces, ventricles, and vascular spaces (all four mice tested; **Figures 2C, Da-g**). Our ability to observe tumor cells even in the left lateral ventricle (**Figure 2Dd**) suggests that this separation was highly unlikely to be caused due to technical reasons, but was instead a result of cell migration; this was also corroborated by the *in vitro* wound-healing assay (**Supplementary Figure 3**). Thus, the H1915 model appeared to closely resemble the clinical features of leptomeningeal carcinomatosis (34–36). The formation of multiple lesions such as leptomeningeal meningitis makes surgical resection impossible, and such patients cannot typically be treated with surgery only (36).

Brain weights of H1915 tumor-bearing mice were increased significantly, with marked enlargement, compared to those of sham-operated mice on day 21 (**Figure 2E**), suggesting that clinical features, including elevated intracranial pressure caused by metastatic brain tumors, were reflected at least in the H1915 model (2, 3). Therefore, these models are likely to provide valuable evaluative tools for predicting the clinical benefits of drug candidates.

### Improvement of Survival Following Treatment of Brain Tumor-Bearing Mice With Perifosine

**Figure 3:**
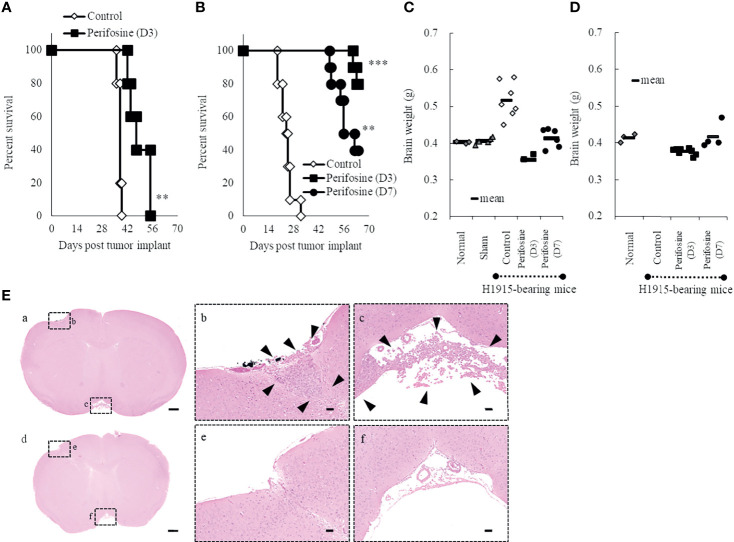
Prolongation of life-span of orthotopic brain metastasis mice with perifosine. Mice were injected intracerebrally with DU 145 **(A)** or H1915 **(B–E)** cells on day 0, and the tumor was allowed to establish until day 3 (perifosine [D3]) or day 7 (perifosine [D7]). Mice were treated with either vehicle or perifosine under the conditions indicated in **Tables 1**, **2**. **(A, B)** Survival curve analysis was performed for 64 days, and the death time point was defined when the animals appeared moribund. **p < 0.01; ***p < 0.001 versus the control group. **(C, D)** The H1915 orthotopic tumor mice were euthanized in a moribund state (**C**; up to day 63) or at the termination of the experiment (**D**; on day 64), and each brain was weighed. Although all 10 mice in the control group were euthanized in a moribund state until day 32, the brains were weighed only for seven mice **(C)**. Age-matched normal (n = 3) and sham-operated (n = 5) mice were included for comparison and euthanized on day 21 **(C)**. **(E)** Representative H&E-stained images of lesions in the H1915 model are shown; scale bar = 500 μm **(a, d)** or 50 μm **(b**, **c**, **e**, **f)**. Low **(a)** and high **(b, c)** power views in the vehicle-treated mouse on day 14 and low **(d)** and high **(e, f)** power views in the perifosine (D3)-treated mouse on day 41. Boxed areas in panels **(a)** and **(d)** show regions depicted in panels **(b, c, e, f)**, respectively. Arrowheads indicate tumor cells.

We used the intracranial DU 145 and H1915 models to evaluate the survival benefit of perifosine against metastatic brain tumors. In the DU 145 model, the MST significantly increased to 49.5 (%ILS = 30, *p* < 0.01) upon administration of perifosine from day 3, while the control group exhibited an MST of 38.0 (**Figure 3A**). In the H1915 model, the MST of the control group was 24.5, while eight out of 10 mice survived until the final day of observation (day 64) in the perifosine (D3) group (MST was not reached, *p* < 0.001). Likewise, treatment with perifosine from day 7 onwards significantly prolonged survival compared to that of the control group, where the MST was 59.0 (%ILS = 141, *p* < 0.01) (**Figure 3B**). Although the survival benefit was lower than that of the perifosine (D3) group, four out of 10 mice survived until day 64 in the perifosine (D7) group. Based on the observation that the control mice began to reach the endpoint as early as on day 19 and the time required for perifosine to reach values close to C_max_ in brain tissues was at least four days after administration (**Figure 1B**), it is noteworthy that perifosine resulted in significant survival benefits, even in the perifosine (D7) group.

Some agents targeting the PI3K/Akt signaling pathway are associated with hyperglycemia due to interaction with the insulin-glucose regulatory axis (37, 38). Therefore, hyperglycemia and polyuria are frequently reported adverse effects of PI3K/Akt inhibitors. Here, the bedding of survivors, all of which were mice in perifosine-treated groups, was visibly wet after day 54, suggesting the symptom of polyuria. In order to assess the possibility of hyperglycemia, mice surviving for 64 days were analyzed for the level of glucose in plasma. As a result, perifosine did not induce an increase in plasma glucose when compared to the historical control data. Similarly, there were no severe changes in T-CHO, T-BIL, BUN, CRE, and ALP on day 64 (**Supplementary Figure 4** and **Supplementary Table 2**).

### Inhibition of H1915 Tumor Growth in the Brain by Perifosine

In the control group of the H1915 model, the brain weight was clearly higher than that in the normal and sham-operated mice, which is suggestive of aggressive tumor progression and/or cerebral edema (**Figures 3C, D**). In contrast, the brain weights in both the perifosine groups were significantly lower than those in the control group, and these values were almost comparable to the values in the normal and sham-operated groups (**Figures 3C, D**).

Tumor cells were observed multifocally in the control group on day 14 (**Figures 3Ea-c**). However, no apparent viable tumor cells were observed in the perifosine (D3)-treated mice on day 41 in a separate experiment (n = 2), thus indicating complete responses (**Figures 3Ed-f**). In agreement with the results of brain weight, it is apparent that perifosine dramatically inhibited H1915 tumor growth.

### Perifosine Preferentially Inhibited the Growth of Ectopic H1915 Xenografts

It has been documented that high correlations between *in vitro* and *in vivo* sensitivity of drugs are not always observed (39–41). Although the exact reason for the discrepancies is unknown, this can be explained, at least in part, by the modification of sensitivity through multiple factors in the tumor microenvironment.

Here, we demonstrated that the growth patterns of DU 145 and H1915 were largely different in brain tissues (**Figures 2A–D**). Therefore, we speculated that the growth patterns of both cell lines in brain tissues may have influenced the *in vivo* sensitivity of perifosine.

**Figure 4:**
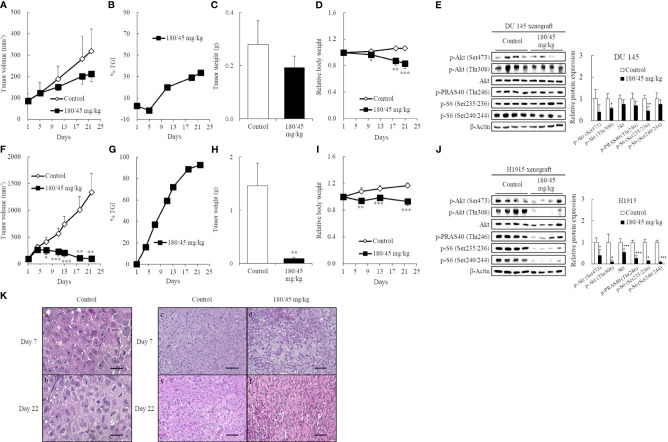
Induction of high antitumor efficacy against ectopic H1915 xenografts in association with blockade of the PI3K/Akt pathway by perifosine. Mice bearing subcutaneous DU 145 **(A-E)** and H1915 **(F-J)** tumors were randomized on day 1 and treated orally with either vehicle or perifosine using a 5-day-on/2-day-off × 3 cycle schedule (n = 5). On day 22, xenograft tumors were excised 4 h following the last dose. Changes in TV **(A, F)**, tumor growth inhibition rate (TGI, %) at different days **(B, G)**, tumor weight **(C, H)**, and relative body weight **(D, I)**. The expression level of key molecules was detected by western blot on Day 22 **(E, J)**. Images are representative of at least two independent experiments with similar results. β-actin was used as a loading control. Protein levels were quantified and represented as the percentage of the control group. *p < 0.05; **p < 0.01; ***p < 0.001 versus the control group. **(K)** Representative H&E images of the H1915-tumor tissues treated with vehicle **(a-c, e)** and perifosine **(d, f)** on days 7 and 22 are shown; scale bar = 20 μm **(a, b)** or 50 μm **(c-f)**. High-power views in the control group suggest that the tumor cells are large, round-to-oval, and possessed distinct multiple nucleoli in the clear oval nucleus **(a**, **b)**.

To confirm this possibility, DU 145 (**Figures 4A–E**) and H1915 (**Figures 4F–K**) cells were subcutaneously inoculated and allowed to form a solitary tumor, and the antitumor effect of perifosine was examined. Perifosine, when used according to the same treatment regimen as was used in the brain orthotopic models, exerted a moderate antitumor effect with a TGI value of 34% in the DU 145 xenograft model, despite the lack of significance [*p* = 0.058 for TV on Day 22 (**Figure 4A**); *p* = 0.086 for tumor weight (**Figure 4C**)]. In accordance with these results, perifosine down-regulated phosphorylation levels of Akt at both Ser473 and Thr308 by 61% (*p* = 0.074) and 44% (*p* < 0.05) respectively without diminishing total Akt protein level in DU 145 xenografts (**Figure 4E**). Meanwhile, perifosine also elicited much stronger antitumor responses against subcutaneous H1915 growth, which included regressions of the established tumor on and after day 7 (93% reduction in TV; **Figures 4F, G**). These results indicated a similar tendency between both *in vitro* cytotoxicity and *in vivo* orthotopic findings, thus demonstrating that perifosine is inherently more potent against H1915 than against DU 145; this was independent of the different growth patterns in brain tissues. Furthermore, perifosine treatment significantly abrogated the Akt signaling pathway in H1915 xenografts on Day 22 (**Figure 4J**), almost consistent with other tumor models (**Supplementary Figure 5**). Notably, both p-Akt and total Akt were reduced at this time point, which is suggestive of an increase in tumor cell death (**Figure 4J**). We also monitored the systemic toxicity of mice receiving this therapy. No adverse effects in general conditions or any treatment-related macroscopic changes in major organs including the brain, liver, and kidneys were induced by this therapeutically effective regimen. Moreover, as the RBW values were more than 0.8, this therapy was deemed tolerable (**Figures 4D, I**).

Histological examination revealed that the tumor cells were large, round-to-oval, and possessed distinct multiple nucleoli in the clear oval nucleus; all of these characteristics resemble the morphological features of large-cell lung carcinoma (**Figures 4Ka, b**). Although there was still no big difference in H1915-tumor volume between the vehicle and perifosine-treated tumors on day 7, we observed marked morphological changes in tumor cells in response to perifosine treatment, which is suggestive of a decrease in tumor cell proliferation and/or an increase in cell death not only on day 22, but also even at this early stage (**Figures 4Kc-f**).

### Perifosine Reduced Cell Proliferation and Induced Apoptosis Preferentially in H1915 Tumor Xenografts

**Figure 5:**
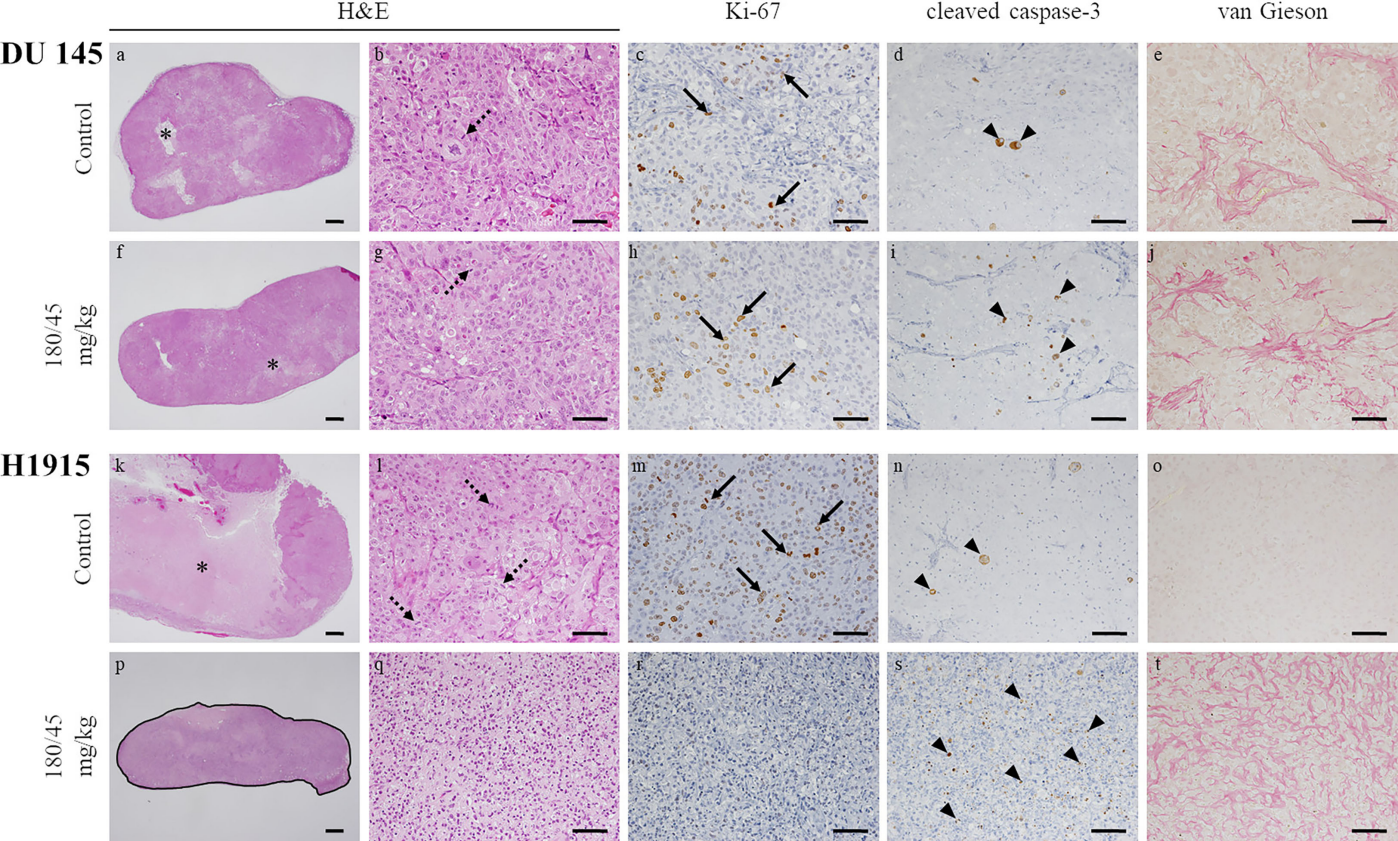
Morphological changes in subcutaneous tumor xenografts evoked by oral administration of perifosine. The resected tumors in **Figures 4C**, **H** were analyzed by H&E staining, immunohistochemical staining for Ki-67 and cleaved caspase-3, and special staining for van Gieson. Representative images of the DU 145 **(A–J)** and H1915 **(K–T)**-tumor tissues treated with vehicle **(A–E, K–O)** and perifosine **(F–J, P–T)** are shown; scale bar = 500 μm **(A, F, K, P)** or 50 μm **(B–E, G–J, L–O, Q–T)**. Granulomatous legion is delineated by a solid black line **(P)**. Perifosine induced granuloma formation around the H1915 tumor lesion **(P)**. Necrosis (asterisks), mitotic figures (dotted arrows), Ki-67-positive cells (arrows), and cleaved caspase-3-positive cells (arrowheads).

To further investigate the mechanism of perifosine-mediated tumor growth inhibition, the levels of Ki-67 and cleaved caspase-3 were assessed in the DU 145 (**Figures 5A–J**) and H1915 (**Figures 5K–T**) tumors. H1915 tumor cells grew into a solitary solid mass, with central necrosis, in the control group on day 22 (**Figure 5K**). Perifosine exhibited a marked decrease in tumor cell proliferation, as detected by Ki-67 and increased apoptosis (cleaved caspase-3 staining), compared to that in tumor cells from the control group, and the majority of the residual tumor tissues treated with perifosine were composed largely of collagen fibers (van Gieson) and well-formed granulomas, which are thought to be formed by the effect of perifosine (**Table 3** and **Figures 5K–T**). Consistently, an increase in the number of tumor cells with apoptotic morphology such as apoptotic body formation and nuclear condensation was observed in the H1915 tumor mass of the perifosine-treated animals (**Table 3**).

**Table 3:** Histopathological evaluation, on day 22, of the tumor tissues treated with perifosine.

Model	Group (n = 5)	necrosis (H&E)					apoptosis: pyknosis/karyorrhexis (H&E)					granuloma (H&E)				
		-	±	1+	2+	3+	-	±	1+	2+	3+	-	±	1+	2+	3+
H1915	Control	0	0	0	4	1	0	5	0	0	0	5	0	0	0	0
	180/45 mg/kg	5	0	0	0	0	0	0	1	4	0	0	0	0	0	5
DU 145	Control	0	4	1	0	0	0	5	0	0	0	5	0	0	0	0
	180/45 mg/kg	0	4	1	0	0	0	2	3	0	0	5	0	0	0	0
**Model**	**Group (n = 5)**	**proliferation (Ki-67)**					**apoptosis (cleaved caspase-3)**					**fibrosis (van Gieson)**				
		**-**	**±**	**1+**	**2+**	**3+**	**-**	**±**	**1+**	**2+**	**3+**	**-**	**±**	**1+**	**2+**	**3+**
H1915	Control	0	0	0	5	0	0	5	0	0	0	4	1	0	0	0
	180/45 mg/kg	3	2	0	0	0	0	0	0	5	0	0	0	0	5	0
DU 145	Control	0	0	5	0	0	0	5	0	0	0	0	0	5	0	0
	180/45 mg/kg	0	1	4	0	0	0	1	4	0	0	0	0	5	0	0

-, absent; ±, minimal; 1+, mild; 2+, moderate; 3+, marked. The results shown in **Figure 5** are quantitatively shown in this table.

Regarding the DU 145 xenografts, although an increase in the number of cleaved caspase-3 positive tumor cells was detected in response to perifosine treatment, there were no obvious differences in the morphological observations, staining patterns of Ki-67, and van Gieson staining between the control and perifosine groups (**Table 3** and **Figures 5A–J**). Special and immunohistochemical stains revealed the changes underlying the antitumor responses to perifosine, thus indicating consistency with the above-mentioned findings.

### Perifosine Preferably Accumulates in H1915 Tumor Xenografts

**Figure 6:**
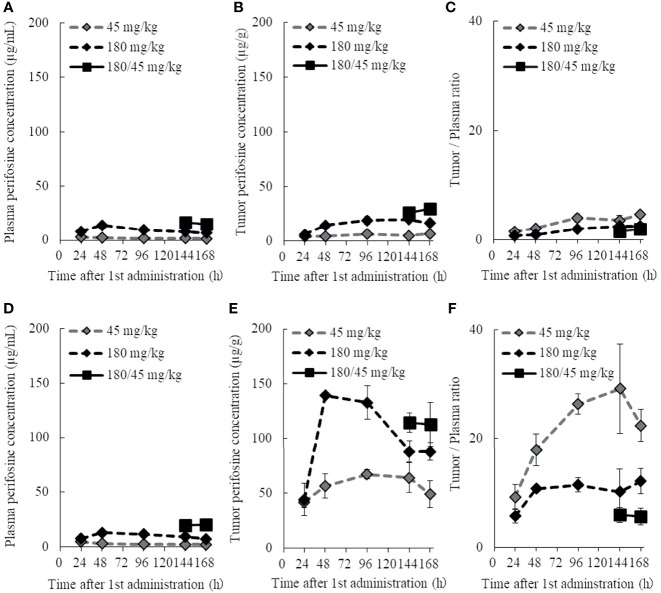
Plasma and tumor pharmacokinetic profiles of perifosine after single and repeated doses. Nude mice with DU 145 **(A–C)** and H1915 **(D–F)** subcutaneous xenograft tumors were randomized and treated with single doses of 45 and 180 mg/kg perifosine or a 5-day repeated dose of 180/45 mg/kg (180 mg/kg *loading* dose followed by *maintenance* doses of 45 mg/kg) perifosine. Plasma **(A, D)** and tumor **(B, E)** samples were collected at the indicated points. Chronological changes in tumor-to-plasma ratios of perifosine are shown for DU 145 **(C)** and H1915 **(F)** models (n = 3, except at 48-h time point for the 180 mg/kg group in **(E, F)**, where n = 2 due to tumor sample processing error).

It has been documented that drug accumulation differs among tumor xenografts (42). Herein, we hypothesized that the different sensitivities to perifosine depend on the differences in drug levels between DU 145 and H1915 xenografts, and characterized the pharmacokinetic properties of perifosine (**Figure 6** and **Supplementary Table 3**).

The plasma concentration of perifosine gradually increased, reached C_max_ at 24 or 48 h after a single dose of 45 or 180 mg/kg, respectively, and then decreased slowly (**Figures 6A, D**). There were no apparent differences in plasma C_max_ and AUC between the DU 145 and H1915 subcutaneous transplantation models. Although the tumor levels of perifosine remained almost stable at 48 h and beyond in both models (**Figures 6B, E**), some differences were observed. In the H1915 tumor xenografts, the AUC of perifosine was 10.7-fold (45 mg/kg) and 6.5-fold (180 mg/kg) higher than that in the DU 145 xenografts; this was corroborated by the data demonstrating that perifosine was favorably delivered into the H1915 tumor tissues, where the tumor-to-plasma ratios were much higher in the H1915 model (**Figures 6C, F**, and **Supplementary Table 3**). These findings indicate H1915-preferential targeting of perifosine and are closely consistent with the above-mentioned results, which showed anti-proliferative activity *in vitro* and antitumor activity in orthotopic and ectopic tumor models were much stronger against H1915 than against DU 145 (**Figures 1D**, **3**, and **4**). Although tumor perifosine concentrations after repeated oral administrations in the 180/45 mg/kg group were higher than those after single doses in the 45 and 180 mg/kg groups, the difference was slight (**Figures 6B, E**).

## Discussion

In the current study, we reported the usefulness of human metastatic brain tumor models and the effectiveness of perifosine in these tumor models. First, we determined that perifosine could distribute into the brain and remain localized there for a prolonged period following oral administration. These data were confirmed in healthy mice with intact BBBs, indicating that perifosine could cross the BBBs. Second, to evaluate the efficacy of perifosine, we established orthotopic xenograft mouse models by injecting the human metastatic brain tumor cell lines DU 145 and H1915 into the brains of these mice. Both models exhibited different apparent growth patterns as compared to those observed using the human glioblastoma cell line U-87 MG. Moreover, multiple tumor lesions were detected remotely from each other in the brain of the H1915 model mice (**Figures 2C, D**, and **3E**), indicating that the surgical treatment would be impractical in clinical settings (3). Third, perifosine significantly increased survival for the DU 145 and H1915 orthotopic brain tumor mice, which was also accompanied by complete regression in the H1915 model (**Figures 3B, E**). Fourth, compared to orthotopic tumors, a similar trend of sensitivity to perifosine was observed when the subcutaneous solitary tumors derived from DU 145 and H1915 were treated with perifosine. These findings suggest that the difference in the sensitivity of perifosine against orthotopic tumors was less likely to be attributable to the difference in the growth pattern in brain tissues, but was instead due to intrinsic differences among the cancers themselves. Finally, we confirmed that the suppression level of H1915-tumor growth was associated with the high accumulation of perifosine at the tumor site and the resultant blockage of the PI3K/Akt signaling pathway, decrease in tumor cell proliferation, and increased apoptosis; these findings suggested that these are all indispensable events underlying the *in vivo* antitumor activity of perifosine.

Perifosine is a bioavailable alkylphospholipid consisting of an 18-carbon alkyl chain. Therefore, we hypothesize that perifosine is taken up into the lipid bilayer of vascular endothelial cells due to the lipophilic characteristics of this molecule. It then penetrates the vascular wall and is gradually transferred into the cerebrospinal fluid over a long period. In contrast, as the brain is an organ with a high lipid level, highly lipophilic perifosine is rapidly transferred from cerebrospinal fluid to brain tissues and accumulates there. As a result, the level of perifosine in the cerebrospinal fluid is always low, and perifosine continues to be transferred from the blood to the cerebrospinal fluid owing to a concentration gradient. Thus, we expect that the unidirectional penetration of perifosine from blood to brain tissues passing through cerebrospinal fluid is continuous, ultimately resulting in long-term retention in brain tissues (**Figures 1A–D**). Additionally, the involvement of flippases and scramblases has been proposed as one possible mechanism of perifosine uptake in cancers (43). It has been documented that flippases drive inward-directed translocation of phospholipids across the plasma membrane, and scramblases transfer lipids from the inner to the outer and from the outer to the inner leaflet of the plasma membrane (44–46). Therefore, flippases and scramblases may be vital for uptake of the alkylphospholipid perifosine by cancer cells and may play a crucial role in modulating the response rate. Although the precise mechanism underlying the accumulation of perifosine in tumor tissues has not yet been fully elucidated, we believe that the enhanced exposure at the target site could enable perifosine to exhibit its antitumor activity.

A research group reported that perifosine monotherapy did not exhibit enough antitumor activity to prolong survival in a genetically engineered mouse model of brainstem gliomas (47). The group described that this may be attributable to poor drug delivery into the brainstem due to the BBBs. However, we administered perifosine at 180/45 mg/kg for nine cycles in the present study, whereas this research group only dosed perifosine at 30 mg/kg for one week. Therefore, we predict that the discrepancy between their data and ours may be due to differences in the experimental settings, as we demonstrated here that perifosine was efficiently transferred into the brain and exhibited encouraging antitumor efficacy.

In this study, we found that perifosine did not induce an increase in plasma glucose, which is one of the on-target adverse events of PI3K/Akt inhibitors. Reportedly, an increase in plasma glucose levels returns to baseline levels within 24 h after an Akt inhibitor AZD5363 treatment, suggesting a transient phenomenon as observed for a PI3K inhibitor copanlisib (38, 48). Therefore, the result here implies that a rise in plasma glucose level, if any, was transient. In our preliminary study, all three mice with H1915 intracranial xenograft tumors that received only six cycles of perifosine similarly survived until the end of the observation period. Based on this, we speculate that we could reduce the dose of perifosine; this research strategy will be employed in our future studies.

Here, we reported that perifosine continued to be retained in the brain for a long period. Although high perifosine concentration in the brain is a vital factor to facilitate therapeutic effects against brain tumors, it could lead to unfavorable side effects associated with CNS disorders. Therefore, it is crucial to determine whether perifosine causes undesirable CNS dysfunction in patients and not just in mice. However, this concern appears to have been recently addressed clinically, where no symptoms associated with CNS disorders were noted in patients, to date, with the exception of manageable toxicities such as anemia, diarrhea, and nausea (49, 50).

Our study does possess a few limitations that are worth noting. Specifically, despite the high degree of homology in the amino acid content of an ATP-dependent efflux transporter, P-glycoprotein in different species and its function may differ across different species (51, 52). It is therefore meaningful for us to examine the species differences, in future studies. Regardless, to our knowledge, our study reports the first evidence of a successful therapy using perifosine against metastatic brain tumors and provides potentially beneficial information for this patient population.

In conclusion, we clearly demonstrated that orally administered perifosine exhibited promising antitumor efficacy in H1915 intracranial orthotopic and ectopic tumor models in association with blockade of the PI3K/Akt pathway. Our results support the concept that long-term exposure to a high level of perifosine at the tumor site and suppression of PI3K/Akt pathway activation are crucial events underlying the *in vivo* antitumor activity of perifosine. Although it is important to validate our findings using clinical specimens, the preclinical evidence presented here reveals a promising future approach for the treatment of patients with metastatic brain cancers and emphasizes the importance of enriching a patient population that has a higher probability of responding to perifosine.

## Data Availability Statement

The original contributions presented in the study are included in the article/**Supplementary Material**. Further inquiries can be directed to the corresponding author.

## Ethics Statement

The animal study was reviewed and approved by the Animal Experimental Committee of the Yakult Central Institute.

## Author Contributions

KT, TS, and AT conceived and designed the study. KT, TS, TO, AK, and MT conducted the experiments and prepared the figures and tables. KT provided data analysis and wrote the original draft. GN, SI, SK, AT, MT, and YS supervised the research. All authors have read and approved the final manuscript.

## Conflict of Interest

KT, TS, TO, AK, GN, SI, SK, AT, MT, and YS are employed by Yakult Honsha Co., Ltd.

## Publisher’s Note

All claims expressed in this article are solely those of the authors and do not necessarily represent those of their affiliated organizations, or those of the publisher, the editors and the reviewers. Any product that may be evaluated in this article, or claim that may be made by its manufacturer, is not guaranteed or endorsed by the publisher.
