# Supplementary material for: Perifosine, a Bioavailable Alkylphospholipid Akt Inhibitor, Exhibits Antitumor Activity in Murine Models of Cancer Brain Metastasis Through Favorable Tumor Exposure
Source: Front Oncol. 2021 Nov 4;11:754365. doi: 10.3389/fonc.2021.754365 (PMC8600181; doi:10.3389/fonc.2021.754365)
Supplement: Supplementary file 1 [file DataSheet_1.docx]

Supplementary Material

**Supplementary TABLE 1 List of primary antibodies, including company catalog numbers.**

| **Western blotting primary antibody** | **Dilution** | **Company and catalog number** |
| --- | --- | --- |
| Rabbit anti-p-Akt (S473) | 1:3000 | Cell Signaling Technologies (No. 4058) |
| Rabbit anti-p-Akt (T308) | 1:1000 | Cell Signaling Technologies (No. 2965) |
| Rabbit anti-Akt | 1:1000 | Santa Cruz Biotechnologies (No. sc-8312) |
| Rabbit anti-p-PRAS40 (T246) | 1:1000 | Cell Signaling Technologies (No. 2640) |
| Rabbit anti-p-S6 (S235/236) | 1:1000 | Cell Signaling Technologies (No. 2211) |
| Rabbit anti-p-S6 (S240/244) | 1:2000 | Cell Signaling Technologies (No. 5364) |
| Mouse anti-cytokeratin | 1:300 | Agilent Technologies (No. M3515) |
| Rabbit anti-cleaved caspase-3 | 1:3000 | Cell Signaling Technologies (No. 9661) |
| Mouse anti-Ki-67 | 1:500 | Thermo Fisher Scientific (No. RM-9106-S0) |
| Mouse anti-β-actin | 1:5000 | Sigma-Aldrich (No. A5316) |

**Supplementary TABLE 2 Blood chemistry measurement in surviving mice on day 64.**

|  | Glucose  (mg/dL) | T-CHO  (mg/dL) | T-BIL  (mg/dL) | BUN  (mg/dL) | CRE  (mg/dL) | ALP  (U/L) |
| --- | --- | --- | --- | --- | --- | --- |
| Perifosine (D3) | 84.2 ± 21.8 | 85.2 ± 17.0 | 0.04 ± 0.01 | 31.3 ± 8.9 | 0.14 ± 0.03 | 247.0 ± 42.1 |
| Perifosine (D7) | 129.3 ± 20.0 | 122.8 ± 17.4 | 0.04 ± 0.01 | 27.0 ± 6.0 | 0.12 ± 0.01 | 359.3 ± 34.2 |
| Historical control data | 128.4 ± 24.2 | 84.7 ± 13.3 | 0.07 ± 0.02 | 17.7 ± 2.2 | 0.08 ± 0.01 | 322.1 ± 36.0 |

The survivors in **Figure 3B**, all of which were mice in the perifosine (D3) and (D7) groups, were analyzed (n = 8 and 4, respectively). On day 64, blood was collected from the abdominal vena cava of all surviving mice under isoflurane anesthesia at 24 h after the final dose. Blood samples were treated with heparin to obtain plasma. Historical control data were included for comparison (n = 10). T-CHO, total cholesterol; T-BIL, total bilirubin; BUN, blood urea nitrogen; CRE, creatinine; ALP, alkaline phosphatase. The values are expressed as mean ± standard deviation (SD).

**Supplementary TABLE 3 Pharmacokinetic profiles of perifosine in plasma and tumor tissues after a single oral dose.**

(**A**)

|  | **Plasma** | | **Tumor** | |
| --- | --- | --- | --- | --- |
| Dose | 45 mg/kg | 180 mg/kg | 45 mg/kg | 180 mg/kg |
| C_max_ (μg/mL or g) | 2.85 | 14.05 | 6.75 | 20.25 |
| T_max_ (h) | 24 | 48 | 168 | 144 |
| AUC (μg·h/mL or g) | 267 | 1420 | 805 | 2447 |
| MRT (h) | 88 | 90 | 99 | 103 |
| T_1/2_ (h) | 336 | 153 | 941 | 424 |

(**B**)

|  | **Plasma** | | **Tumor** | |
| --- | --- | --- | --- | --- |
| Dose | 45 mg/kg | 180 mg/kg | 45 mg/kg | 180 mg/kg |
| C_max_ (μg/mL or g) | 4.81 | 13.04 | 67.17 | 139.11 |
| T_max_ (h) | 24 | 48 | 96 | 48 |
| AUC (μg·h/mL or g) | 402 | 1531 | 8625 | 15844 |
| MRT (h) | 88 | 92 | 98 | 94 |
| T_1/2_ (h) | 314 | 111 | 177 | 113 |

Data were calculated based on the mean values of pharmacokinetic parameters in both subcutaneous DU145 (**A**) and H1915 (**B**) tumor xenograft models. The AUC and MRT up to the last sampling point (168 h) were calculated (0-last).

**
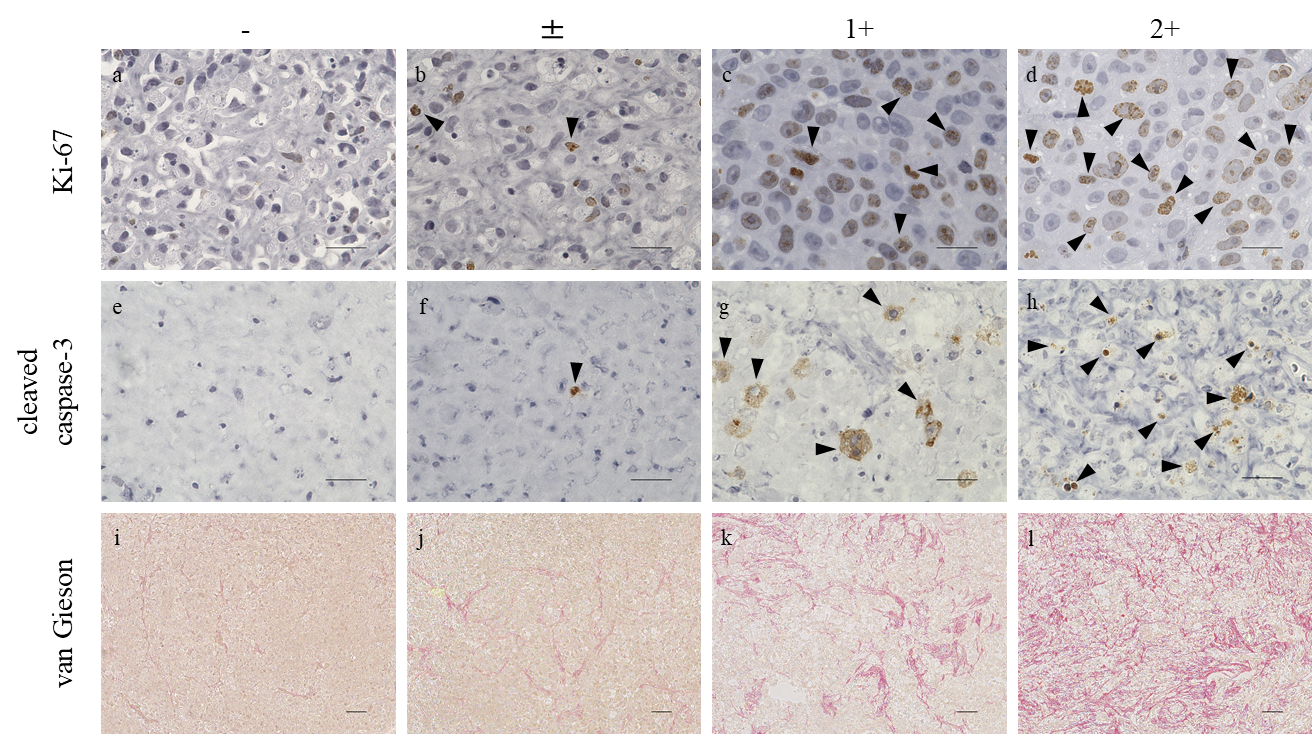
**

**Supplementary FIGURE 1** **Evaluation and scoring of Ki-67, cleaved caspase-3, and van Gieson images.**

Representative images taken from tumor tissues with various levels of Ki-67 (**a**-**d**), cleaved caspase-3 (**e**-**h**), and van Gieson (**i**-**l**) expressions are shown. We examined at least five high power fields (×200) in five different areas of each histological section and calculated the average number of positive tumor cells (**a**-**h**). The Ki-67 was defined as the percentage of tumor cells positively stained in the examined field(s): -, absent; ±, fewer than one-quarter of cells per each field; 1+, one-quarter to one-half of cells per each field; 2+, one-half to three-quarters of cells per each field (**a**-**d**). The cleaved caspase-3 score was categorized as follows: -, absent; ±, fewer than 25 cells per each field; 1+, 25-250 cells per each field; 2+, more than 250 cells per each field (**e**-**h**). We also evaluated collagen fibers stained red on van Gieson staining (**i**-**l**): -, absent; ±, focal positive of collagen with no bundles; 1+, focal bundles of collagen, occasionally with intersections; 2+, diffuse and dense bundles of collagen with extensive intersections around the lesion (**i**-**l**). Scale bar = 20 μm (**a**-**h**) or 50 μm (**i**-**l**). Arrowheads indicate positive cells.


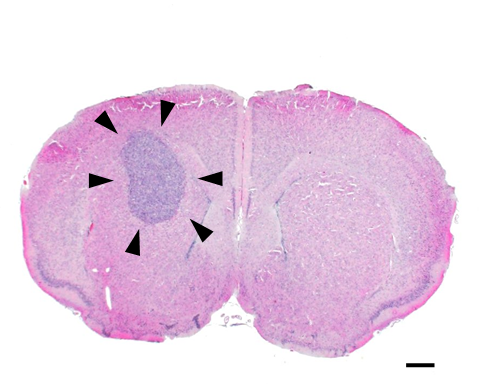


**Supplementary FIGURE** **2 Representative morphological feature of U-87 MG tumor cells following implantation.**

Mice were injected intracerebrally with the primary glioblastoma U-87 MG cells (1 × 10^6^ viable cells in 5 μL PBS) on day 0, and the tumor was allowed to establish until day 14. Tumor region is indicated by black arrows. Representative H&E-stained image of the brain is shown. Scale bar = 500 μm.


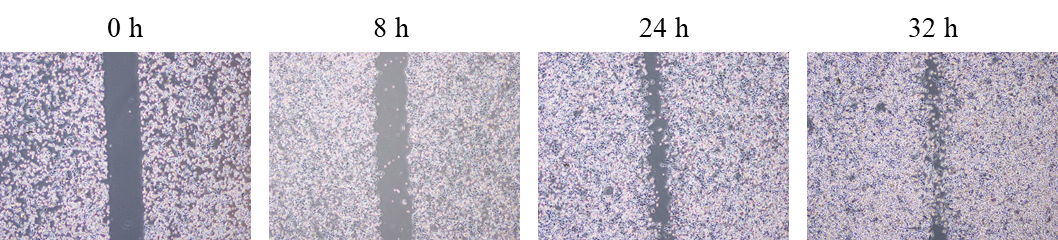


**Supplementary FIGURE 3 Analysis of cell migration by *in vitro* wound-healing assay.**

The migration and invasion abilities of H1915 cells were evaluated based on the wound-healing assay using the ibidi Culture-Inserts. Cells were seeded on both sides of the 2-well culture inserts (ibidi, Germany) and incubated overnight to reach confluence. Representative microscopy images of wound closure of H1915 cells at 0, 8, 24, and 32 h after culture insert removal are shown. The images are representative of two independent experiments.


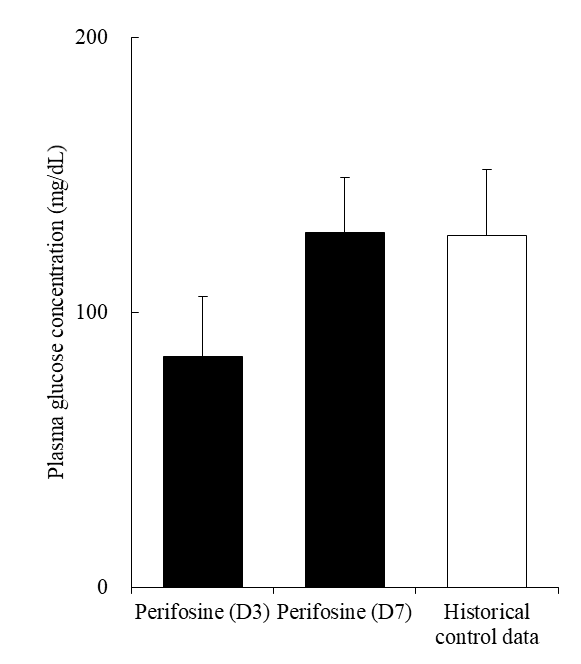


**Supplementary FIGURE 4 Analysis of plasma glucose concentration on day 64.**

The survivors in **Figure 3B**, all of which were mice in the perifosine (D3) and (D7) groups, were analyzed (n = 8 and 4, respectively). On day 64, blood was collected from the abdominal vena cava of all surviving mice under isoflurane anesthesia at 24 h after the final dose. Plasma glucose levels were analyzed. Historical control data were included for comparison (n = 10).


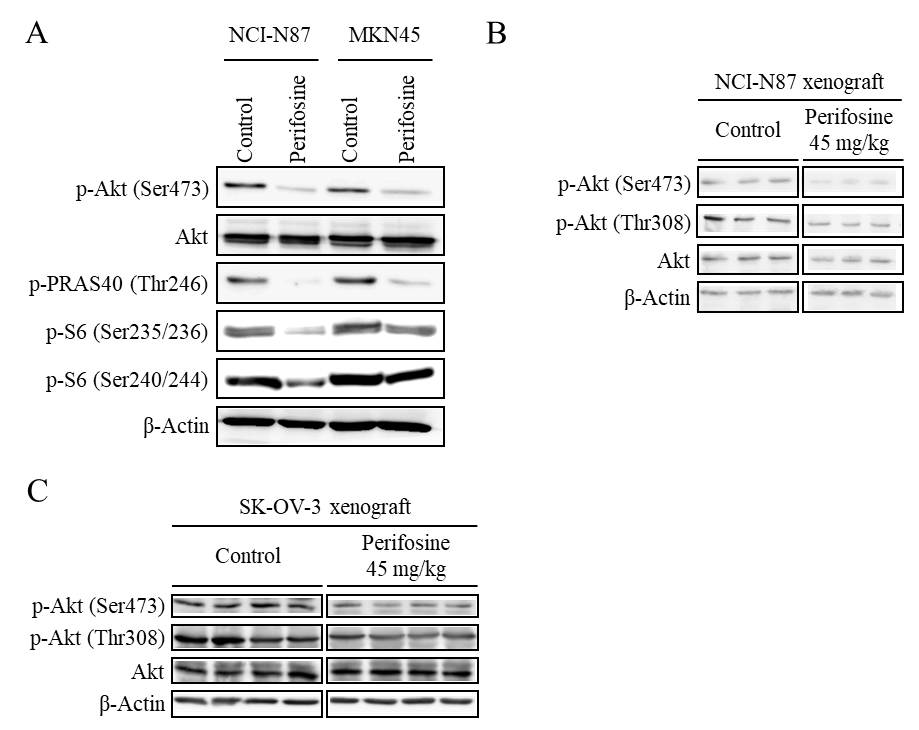


**Supplementary FIGURE 5 Analysis of change in crucial molecules in the PI3K/Akt pathway in tumors.**

Phosphorylation and activation of key molecules in the PI3K/Akt signaling pathway were examined with western blotting. Human gastric cancer NCI-N87 and MKN45 cells were left untreated or treated with 15 μM perifosine for 16 h. The blots were probed with the respective primary antibodies (**A**). Mice bearing subcutaneous NCI-N87 tumors were randomized on day 1 when the tumors reached an average volume of 100 mm^3^ and treated orally with either vehicle or perifosine (45 mg/kg) for 5 days (**B**). Mice were injected intraperitoneally with human ovarian cancer SK-OV-3 cells (1 × 10^7^ viable cells in 0.5 mL PBS) and assigned homogeneously to each test group based on the body weight three days later (day 1). The animals were treated orally with either vehicle or perifosine (45 mg/kg) with a 5-day-on/2-day-off schedule (**C**). Lysates were prepared from NCI-N87 and SK-OV-3 xenografts 4 h after the last dose on days 5 and 51, respectively (**B**, **C**). Western blot images are representative of at least two independent experiments with similar results. β-actin was used as a loading control.
